# Socioeconomic inequalities in HIV knowledge, HIV testing, and condom use among adolescent and young women in Latin America and the Caribbean

**DOI:** 10.26633/RPSP.2021.47

**Published:** 2021-05-26

**Authors:** Juan Pablo Gutiérrez, Alejandra Trossero

**Affiliations:** 1 National Autonomous University of Mexico (UNAM) Mexico City Mexico National Autonomous University of Mexico (UNAM), Mexico City, Mexico; 2 UNICEF Regional Office for Latin America and the Caribbean Panama Panama UNICEF Regional Office for Latin America and the Caribbean, Panama, Panama

**Keywords:** Adolescent health, sexual and reproductive health, health inequality monitoring, HIV, sexually transmitted diseases, Latin America, Caribbean Region, Salud del adolescente, salud sexual y reproductiva, monitoreo de las desigualdades en salud, VIH, enfermedades de transmisión sexual, América Latina, Región del Caribe, Saúde do adolescente, saúde sexual e reprodutiva, monitoramento das desigualdades em saúde, HIV, doenças sexualmente transmissíveis, América Latina, Região do Caribe

## Abstract

**Objective.:**

To appraise the presence and magnitude of inter- and intra-country health inequalities related to HIV in Latin America and the Caribbean (LAC) among young females.

**Methods.:**

We analyzed household surveys in twenty LAC countries, that included data from female adolescents and young women (ages 15-24) between 2008 and 2018, measuring inequality with the concentration index of 4 indicators: 1) whether individuals have heard of HIV or not, 2) a composite variable of correct knowledge, 3) reported condom use with the last partner, and 4) whether individuals were ever tested for HIV.

**Results.:**

Participants from households in countries with higher socioeconomic status are more likely to have heard of HIV, have correct knowledge of HIV transmission, and have used condoms during their last sexual intercourse. The inter-country concentration index for those indicators were 0.352, 0.302 and 0.110, respectively.

**Conclusions.:**

Economically disadvantaged female adolescents and young women in LAC face an increased risk for HIV, as they are less aware of HIV and its actual transmission mechanism and are less likely to use condoms with their sexual partners. There is an urgent need to tailor prevention strategies of sexually transmitted infections and HIV for adolescents and young women that are sensitive to their socioeconomic context.

## RESUMEN

**Objetivo.:**

Evaluar la presencia y la magnitud de las desigualdades en la salud entre los países y dentro de cada país en relación con la infección por el VIH en las mujeres jóvenes en América Latina y el Caribe.

**Métodos.:**

Analizamos encuestas de hogares en veinte países de América Latina y el Caribe con datos sobre las adolescentes y las mujeres jóvenes (de edades entre 15 y 24 años) entre el 2008 y el 2018. En estas encuestas se medía la desigualdad con un índice de concentración de cuatro indicadores: 1) si sabían lo que era el VIH o no, 2) una variable compuesta con respecto a los conocimientos correctos, 3) si habían usado preservativo con su pareja más reciente, y 4) si se habían hecho alguna vez la prueba del VIH.

**Resultados.:**

Las participantes de los hogares en países con una situación socioeconómica más alta tienen mayores probabilidades de saber acerca del VIH, de tener los conocimientos correctos con respecto a la transmisión del VIH y de haber usado preservativo en su relación sexual más reciente. El índice de concentración entre países para esos indicadores fue de 0,352, 0,302 y 0,110, respectivamente.

**Conclusiones.:**

Las adolescentes y las mujeres jóvenes económicamente desfavorecidas en América Latina y el Caribe se enfrentan a un riesgo mayor de contraer el VIH, ya que saben menos sobre este virus y su mecanismo real de transmisión, y es menos probable que usen preservativo con sus parejas sexuales. Hay una necesidad urgente de adaptar las estrategias de prevención de las infecciones de transmisión sexual y de la infección por el VIH para las adolescentes y las mujeres jóvenes que son susceptibles a su contexto socioeconómico.

## RESUMO

**Objetivo.:**

Avaliar a presença e a dimensão das desigualdades em saúde relacionadas ao HIV entre os países e dentro de cada país em adolescentes e mulheres jovens da América Latina e Caribe.

**Métodos.:**

Analisamos pesquisas domiciliares realizadas em 20 países da América Latina e Caribe contendo dados de adolescentes do sexo feminino e mulheres jovens (15 a 24 anos) para o período entre 2008 e 2018, mensurando a desigualdade pelo índice de concentração segundo 4 indicadores: 1) ter ouvido falar de HIV, 2) uma variável composta de conhecimento correto, 3) uso de preservativo com o último parceiro (autorrelatado) e 4) ter feito o teste de HIV.

**Resultados.:**

As participantes domiciliadas em países com nível socioeconômico mais alto têm uma chance maior de ter ouvido falar de HIV, de ter conhecimento correto da transmissão do vírus e de ter feito uso de preservativos na última relação sexual. Os índices de concentração entre os países para estes indicadores foram 0,352, 0,302 e 0,110, respectivamente.

**Conclusões.:**

As adolescentes e mulheres jovens da América Latina e Caribe em situação econômica menos favorecida têm um risco maior de se infectar pelo HIV porque sabem menos sobre o vírus e seu real mecanismo de transmissão, e é menos provável que façam uso de preservativos com seus parceiros sexuais. As estratégias de prevenção de infecções sexualmente transmissíveis e do HIV precisam urgentemente ser adaptadas ao contexto socioeconômico em que se inserem as adolescentes e mulheres jovens da Região.

Latin America and the Caribbean has been described as the most unequal region in the world, with structural characteristics that have contributed to perpetuating inequities, thus constituting a barrier for development that may be eased by increasing access to public services – in particular health services – for the most vulnerable populations (1).

In terms of the HIV pandemic during the past decade, the world saw a reduction in the number of new HIV infections – (23 % decrease between 2000 and 2019). In Latin America, however there was an increase of 21 % during the same time period. The Caribbean region presents a different pattern with 29 % decrease in new infections over the past decade (2).

In 2019, 17 % of new infections in Latin America occurred in young people (15 to 24 years) – (6 % in young women and 11 % in males). In the Caribbean, 30 % of total new infections occurred in young people (16 % in young women and 14 % in young males) (2). Although there is free access to HIV treatment in most countries in the region, relevant gaps remain in terms of access to testing. The lack of age disaggregated data on HIV prevalence, incidence and access to key HIV services represents a clear limitation to develop strategies to differentially address this challenge by age-groups.

The dramatic changes that take place during adolescence, involving complex interactions of emotional, social, psychological and neuro-developmental processes (3) increases their vulnerability and exposure to risk, particularly to sexually transmitted infections (STI) including HIV (4, 5). While there is insufficient data on STI prevalence among adolescents and young women in the region, from the few existing studies the estimation of chlamydia prevalence is between 7 % to 31 % (6–8) raising an important alert on the synergies of STI and HIV transmission in young people. Fertility rate has clearly declined among all age groups in LAC except in adolescent girls (ages 15-19) (66.5 per 1 000 adolescent girls during 2010-2015). Therefore, adolescent pregnancies account for a large proportion of total births in the region – 15 % of pregnancies occur in women before the age of 20 (9), implying a less than optimal condom use. Evidence also shows that young women from poor, rural, and indigenous communities have an increased likelihood of becoming pregnant during adolescence and being in early unions or married (10). The delivery of integrated services, that could address the sexual and reproductive health needs of young women, is not a reality in those regions, which could be due to fragmentation in existing programs and services (11).

Health inequalities have been described in the region for other relevant outcomes, including stunting, obesity among adults, and contraceptive use (12–14). While recent data describes a pattern for reducing the gap between socioeconomic population quintiles there is still evidence of intra-country inequalities. There are no previous analyses for inequalities on HIV-related indicators among adolescents and young people in the region. While analyses at country level have reported no difference between female adolescents from low and high income households on the age of sexual initiation, condom use in the first sexual intercourse was higher among those from higher income. In Mexico, for example, there is a percentage gap of 20 points in condom use between female adolescents from the bottom and top income quintiles (15).

Comprehensive interventions to address adolescent behaviors must consider the different socioecological levels (environment, community, family, individual), the particular context and the inequalities in access to goods and services related to health, and therefore health outcomes, as a path to effectively reduce new HIV cases in the region (16).

In that sense, the overall aim of this study is to appraise the presence and magnitude of inter- and intra-country health inequalities in Latin America and the Caribbean among females 15 to 24 years old with regard to HIV knowledge, HIV testing and condom use.

## METHODS

An ecological epidemiological analysis was conducted, based on secondary analysis of a set of household surveys in the region, estimating the concentration index (CI) as a measure of health inequality for variables related to HIV risk among females 14 to 24 years old.

### Data

We analyzed probabilistic household surveys available for twenty countries in the region, using UNICEF´s Multiple Indicators Cluster Surveys (MICS) and the Demographic and Health Surveys (DHS). In table 1, we report a list of countries included as well as type of survey, year and sample size in the age range 15 to 24. Both surveys (MICS and DHS) are representative of country population and use standard procedures for data collection. Details on the MICS and DHS methodologies are published elsewhere (17, 18). Ethical approval for each survey was obtained for each country as well as informed consent. For adolescents, informed consent was obtained from a parent or guardian (19).

**TABLE 1.** Countries included in the analysis with program, survey year, and total number of observations from females 10 to 24 years

Country	Program	Year	Total observations
Argentina	MICS	2011	8 158
Barbados	MICS	2012	388
Belize	MICS	2015/16	1 771
Bolivia	DHS	2008	6 335
Colombia	DHS	2015	15 139
Costa Rica	MICS	2011	1 720
Cuba	MICS	2010/11	2 740
Dominican Republic	MICS & DHS	2013 & 2014	11 691
El Salvador	MICS	2014	5 102
Guatemala	DHS	2014/15	10 598
Guyana	MICS & DHS	2009 & 2014	3 666
Haiti	DHS	2012	6 272
Honduras	DHS	2011/12	9 347
Mexico	MICS	2015	4 071
Panama	MICS	2013	3 286
Paraguay	MICS	2016	2 641
Peru	DHS	2010	7 766
Santa Lucia	MICS	2012	402
Surinam	MICS	2018	2 256
Uruguay	MICS	2012/13	760
TOTAL			104 109

***Source:*** Authors based on UNICEF Multiple Indicator Cluster Surveys (MICS) & Demographic and Health Surveys Program (DHS) surveys

For both MICS and DHS, while there are some specificities on the sampling approach in each country, there is a multi-stage cluster sampling with enumeration areas from census data as a primary sampling units and households as secondary sampling units. All women 15 to 49 in the identified household are selected (18, 20).

MICS collects health-related data for children, adolescents and women, including specific questions on HIV knowledge and related behaviors. The DHS survey also includes questions on HIV knowledge and related behaviors. Questionnaires for MICS and DHS are standardized instruments, comparable across countries and have been harmonized between them.

For the purpose of this analysis, we have only included data for females age 15-24, from both MICS and DHS surveys conducted in LAC between 2008 and 2018. Only countries that included HIV related questions in their surveys were considered.

We used the latest available results for countries with at least one survey in the 10-year period. We included data from 14 countries with MICS (Argentina, Barbados, Belize, Costa Rica, Cuba, El Salvador, Guyana, Mexico, Panama, Paraguay, Dominican Republic, Saint Lucia, Surinam, and Uruguay) and 8 countries with DHS (Bolivia, Colombia, Guatemala, Guyana, Haiti, Honduras, Peru and Dominican Republic). For two countries with both MICS and DHS (Guyana and Dominican Republic), we pooled the data from both surveys. A total of 104 109 women were included in the final analysis, although total numbers vary for each indicator.

The list of countries (with survey available) and survey program and year is presented in table 1. While the estimated total size of the population of females age 15 to 24 in the region was 53.5 million for 2015 in 35 countries, in the 20 countries included in this analysis lives about 57 % of the females in the included age range, or 30.3 million.

### Measuring inequalities

To measure inequalities in access to HIV prevention, we used two knowledge indicators and two behavior indicators. For knowledge, we used whether individuals have heard of HIV or not. This is a common question in both MICS and DHS, and it is a “yes or no” question. In addition, we generate a composite variable of correct knowledge of HIV with 4 questions available in both MICS and DHS surveys –knowing that condom use and having a single sexual partner prevents transmission, that HIV is not transmitted by mosquito bites, and that sharing food is not a risk for HIV transmission –, generating a 0 to 4 scale with the highest value indicating adequate knowledge.

For behavior we analyzed reported condom use with the last sexual partner, and whether individuals were ever tested for HIV, both as dichotomous variables with “no” and “yes” options.

We estimated intra-country health inequalities using the wealth index available in both survey programs that is based on households’ assets and dwelling characteristics, a continuous variable with larger values indicating better off households. The DHS wealth index includes all household assets and utility services that are available in a particular survey (21); similarly, the MICS equivalent index includes the set of assets and services available on surveys (22). For the analysis, it is not relevant that variables included for each country/survey program are not identical, as far as the particular index captures socioeconomic heterogeneity in each country. To be able to compare between countries, we normalized the wealth index by country and then scaled using the per-capita gross domestic product adjusted by purchase power parity (pc-GDP-PPP) using data from the World Bank (https://data.worldbank.org/indicator/NY.GDP.PCAP.CD) in order to have a homogeneous measure across countries.

### Analysis

We estimated the concentration index (CI) for each indicator both for the region (inter-country) and for each country (intra-country). The CI is derived from a concentration curve of the health indicator and the population ordered by a socioeconomic indicator. In this case, the household wealth index. The CI is defined as twice the area between the concentration curve and the line of equality (the 45-degree line), and ranges from –1 to 1, where the zero value is an indication of absence of inequality. For the indicators in this analysis, positive values indicate pro-rich indicators, that is, wealthier individuals (countries) are better off in terms of HIV knowledge and related behavior (23, 24).

The CI is estimated by:

$$\mathrm{CI}=\frac{2}{N^{2}\times \mu_{h}}\times \sum_{i}^{n}h_{i}\times r_{i}$$

where *h_i_* is the health indicator, *μ_h_* the mean of the health indicator and *r_i_* is the fractional rank of individual *i* in the wealth index, being the N individual the wealthiest one. As has been discussed by Wagstaff (25), for health indicators that are bounded – such as those analyzed here – the estimates for the standard CI are not bound between –1 and 1. To address this, a normalization has been proposed dividing the CI by 1 minus the mean (26):

$$\mathrm{CI}=\frac{\frac{2}{N^{2}\times \mu_{h}}\times \sum_{i}^{n}h_{i}\times r_{i}}{1-\mu_{h}}$$

For this analysis we estimated the Wagstaff adjusted CI. The CI can be estimated by regression (27) and in particular we used the implementation of this approach in the *conindex* command in Stata (28).

In order to make the estimations comparable across countries, we normalized surveys weights by country and then scaled those values using the relative size of the female population 15 to 24 years old across countries.

In general, it has been suggested a [0.2] threshold in the value of a CI as indicator of a relevant degree of health inequality (29).

We implemented all the analyses using Stata 15 (Stata Corp.).

### Ethical approval and consent to participate

This a secondary analysis of publicly available and anonymized surveys. No primary data was collected for this analysis. Ethical approval for the analyzed surveys were obtained in the respective countries and permission to analyze the data from the selected countries granted by DHS Program and MICS-UNICEF.

## RESULTS

In table 2 we report the average value of the indicators by country. Average age of adolescent girls and young women included in the surveys was similar across countries.

In terms of knowledge of HIV and AIDS, most of the respondents have heard of HIV or AIDS before the surveys, with values above 96 % for most countries, except Belize, Bolivia, Guatemala, Paraguay, and Suriname, where the percent of participants that have heard of HIV/AIDS was 90.89 %, 85.43 %, 88.78 %, 94.25 % and 93.59 %, respectively.

Correct knowledge of HIV/AIDS is heterogeneous across countries; in the scale of 0 to 4, averages range from 1.77 to 3.67 (Guatemala and Barbados, respectively). This variation on correct answers reflects widespread misconceptions about transmission mechanisms, where a relevant percent of individuals believes, for example, that mosquito bites can transmit HIV. As the data from Dominican Republic and Guyana suggests, it is possible that some variations are related to the type of survey (DHS or MICS), although as described in the methodology, procedures for both surveys are quite similar. It is more likely that these variations reflect changes in perceptions among the population.

**TABLE 2.** Average (95 % confidence interval) age and HIV knowledge and related behavior indicators by country for females 15 to 24 years

Country	Age mean	Have heard of AIDS %	HIV knowledge (0 to 4 scale) mean	HIV test %	Condom use last partner %
Argentina	19.32	97.20	3.35	26.64	NA
	(19.20 - 19.45)	(96.50 - 97.90)	(3.31 - 3.40)	(24.15 - 29.13)	
Barbados	19.39	99.82	3.67	36.55	56.66
	(19.10 - 19.69)	(99.47 - 100.17)	(3.60 - 3.74)	(31.03 - 42.06)	(50.55 - 62.78)
Belize	19.29	90.89	3.04	35.96	33.51
	(19.14 - 19.43)	(88.20 - 93.58)	(2.93 - 3.15)	(32.59 - 39.33)	(28.96 - 38.07)
Bolivia	19.10	85.43	2.59	NA	NA
	(19.02 - 19.19)	(83.77 - 87.10)	(2.52 - 2.66)		
Colombia	18.46	97.21	3.07	30.72	30.28
	(18.37 - 18.55)	(96.84 - 97.59)	(3.04 - 3.10)	(29.40 - 32.05)	(28.69 - 31.87)
Costa Rica	19.53	98.93	3.27	23.08	33.81
	(19.31 - 19.75)	(98.12 - 99.75)	(3.17 - 3.38)	(18.63 - 27.52)	(29.62 - 38.00)
Cuba	19.66	99.77	3.52	48.80	54.24
	(19.44 - 19.88)	(99.34 - 100.20)	(3.46 - 3.58)	(43.74 - 53.86)	(49.27 - 59.22)
Dominican Republic (MICS)	19.48	98.99	3.31	37.78	26.55
	(19.39 - 19.57)	(98.73 - 99.26)	(3.28 - 3.34)	(35.97 - 39.59)	(24.57 - 28.54)
Dominican Republic (DHS)	19.03	98.28	2.39	56.73	20.89
	(18.83 - 19.23)	(97.15 - 99.41)	(2.33 - 2.46)	(52.93 - 60.53)	(16.45 - 25.33)
El Salvador	19.23	98.66	3.01	29.19	21.66
	(19.12 - 19.34)	(98.19 - 99.12)	(2.97 - 3.05)	(27.33 - 31.06)	(19.43 - 23.89)
Guatemala	19.19	88.78	1.77	24.13	10.87
	(19.13 - 19.26)	(87.23 - 90.32)	(1.72 - 1.82)	(22.73 - 25.53)	(9.55 - 12.18)
Guyana (MICS)	19.17	97.31	3.38	39.47	36.35
	(19.00 - 19.34)	(96.08 - 98.53)	(3.31 - 3.45)	(35.71 - 43.23)	(31.91 - 40.79)
Guyana (DHS)	19.09	97.33	2.58	47.45	32.15
	(18.94 - 19.24)	(96.19 - 98.46)	(2.53 - 2.64)	(44.42 - 50.48)	(27.67 - 36.62)
Haiti	19.26	99.64	2.26	36.05	36.52
	(19.16 - 19.35)	(99.45 - 99.84)	(2.23 - 2.29)	(34.16 - 37.94)	(33.78 - 39.26)
Honduras	19.20	96.65	3.07	38.47	13.61
	(19.12 - 19.27)	(96.15 - 97.14)	(3.04 - 3.10)	(37.08 - 39.85)	(12.21 - 15.02)
Mexico	19.53	96.14	3.01	21.49	32.15
	(19.35 - 19.72)	(94.88 - 97.40)	(2.95 - 3.08)	(18.38 - 24.60)	(28.68 - 35.63)
Panama	19.33	96.45	3.04	29.50	25.76
	(19.14 - 19.52)	(95.30 - 97.61)	(2.95 - 3.12)	(26.22 - 32.78)	(21.66 - 29.87)
Paraguay	19.32	94.25	2.81	28.18	45.84
	(19.16 - 19.47)	(92.92 - 95.58)	(2.74 - 2.89)	(25.51 - 30.84)	(42.33 - 49.35)
Peru	19.11	96.06	2.03	78.59	19.34
	(19.02 - 19.20)	(95.32 - 96.80)	(2.00 - 2.06)	(77.11 - 80.07)	(17.35 - 21.32)
Saint Lucia	19.31	98.89	3.58	41.13	52.01
	(19.01 - 19.60)	(97.61 - 100.17)	(3.50 - 3.66)	(35.87 - 46.39)	(45.12 - 58.89)
Surinam	18.99	93.59	3.15	25.56	24.23
	(18.83 - 19.14)	(92.04 - 95.13)	(3.08 - 3.23)	(22.76 - 28.37)	(21.43 - 27.03)
Uruguay	19.17	98.46	3.40	26.40	57.53
	(18.71 - 19.63)	(97.04 - 99.88)	(3.29 - 3.51)	(19.13 - 33.67)	(46.29 - 68.76)
Observations	104,108	104,106	104,108	84,595	51,968

***Source:*** Authors based on results obtained analyzing UNICEF Multiple Indicator Cluster Surveys (MICS) & Demographic and Health Surveys Program (DHS) surveys NA: not available.

There is also an important inter-country variation on HIV testing among female adolescents and youths. The percent of those 15 to 24 years old that have been tested for HIV ranges from 21.49 % in Mexico in 2015 to 78.59 % in Peru in 2010.

Also, there was a large inter-country variation on reported condom use during the last sexual intercourse. At the bottom, the percentage of adolescents and young females in Guatemala using condoms in their last sexual intercourse was 10.87 %; compared to 57.53 % among adolescent and youth females in Uruguay. Nevertheless, it is surprising that even in the country with the largest percent of condom use, close to half of women 15 to 24 did not use condom in their last sexual intercourse. Overall, it seems that young women in countries reporting higher probability of having heard of AIDS and had greater knowledge of HIV were also more likely to use condoms, while HIV testing does not seem to be related to greater condom use.

### Concentration index

The region as a whole, has a pro-rich distribution in terms of knowledge of HIV/AIDS, as we report in the first row of table 3, with a CI of 0.352, that is, a large magnitude on the inequality. Correct knowledge of HIV transmission is also pro-rich, with a CI of 0.302, indicating that in those countries with higher per capita GDP-PPP, individuals are more likely to know about HIV/AIDS and to have correct information about it, in particular regarding transmission mechanisms.

Testing for HIV is not different in terms of GDP-PPP per capita between countries in the region. Condom use in the last sexual intercourse is also pro-rich, with a CI of 0.110, i.e., more women in countries with higher per capita GDP-PPP reporting condom use in their most recent sexual intercourse.

In table 3 we present the CI for all the 20 countries in the analysis for each of the 4 variables to measure intra-country inequalities. It´s important to mention that for HIV testing and condom use in the last sexual intercourse, there is no data available for Argentina and Bolivia.

**TABLE 3.** Concentration index as a measure of health inequalities for HIV/AIDS related indicators using household wealth as economic stratifier, by country

Country	Have heard of AIDS	Correct knowledge of HIV	HIV test	Condom use with last partner
LAC	0.352	0.302	NS	0.110
Argentina	0.447	0.205	NA	NA
Barbados	NS	NS	–0.135	NS
Belize	0.509	0.303	0.072	0.295
Bolivia	0.667	0.425	NA	NA
Colombia	0.656	0.155	–0.162	0.244
Costa Rica	0.586	0.327	–0.160	0.136
Cuba	NS	0.050	0.156	0.063
Dominican Republic	0.587	0.220	–0.160	0.230
El Salvador	0.450	0.217	NS	0.201
Guatemala	0.627	0.249	0.134	0.444
Guyana	0.447	0.120	NS	0.165
Haiti	0.448	0.083	0.139	0.330
Honduras	0.650	0.279	–0.046	0.317
Mexico	0.558	0.186	0.140	0.262
Panama	0.822	0.325	0.058	0.223
Paraguay	0.480	0.255	NS	0.292
Peru	0.757	0.145	0.381	0.383
Saint Lucia	NS	0.181	NS	NS
Surinam	0.452	0.245	–0.222	NS
Uruguay	0.614	0.199	–0.285	0.279

***Source:*** Authors based on results obtained analyzing UNICEF Multiple Indicator Cluster Surveys (MICS) & Demographic and Health Surveys Program (DHS) surveys

NS: not statistically significantly different from zero at 5 % level; NA: not available

Using the per capita GDP-PPP adjusted wealth index, there is an important heterogeneity in the inequalities in the region. For the variable of *having heard of AIDS*, the CI goes from not statistically significantly different from zero (Cuba and Barbados), to 0.822 in Panama and 0.757 in Peru. It is important to highlight that for 18 of the 20 analyzed countries there is a pro-rich distribution of this indicator; individuals from wealthier households are more likely to have heard of AIDS.

The correct knowledge of HIV is unequally distributed in 19 of the 20 countries in this analysis, being Barbados the only exception. For all the 19 countries with CI values statistically different from zero, the distribution is pro-rich, that is, higher values for those with higher wealth index. Cuba presents the lowest significant CI for this indicator (0.050), which goes up to 0.327 in Costa Rica and 0.425 in Bolivia.

HIV testing presents a differential pattern: 6 countries have a pro-poor distribution (higher for those with lower wealth index), and 8 countries present a pro-rich distribution. For El Salvador, Guyana, Paraguay, and Saint Lucia, the estimated CI is not different from zero. The larger CI for the pro-poor group is Uruguay with a CI of –0.285, while the larger CI for the pro-rich group is Peru with a CI of 0.381.

Finally, reported condom use in the last sexual intercourse in the 18 countries with data available for this variable (excluding Argentina and Bolivia), in all but two (Saint Lucia and Surinam), there is a pro-rich distribution. The lowest CI for these 16 countries is 0.063 in Cuba, and the largest 0.444 in Guatemala.

**FIGURE 1.**
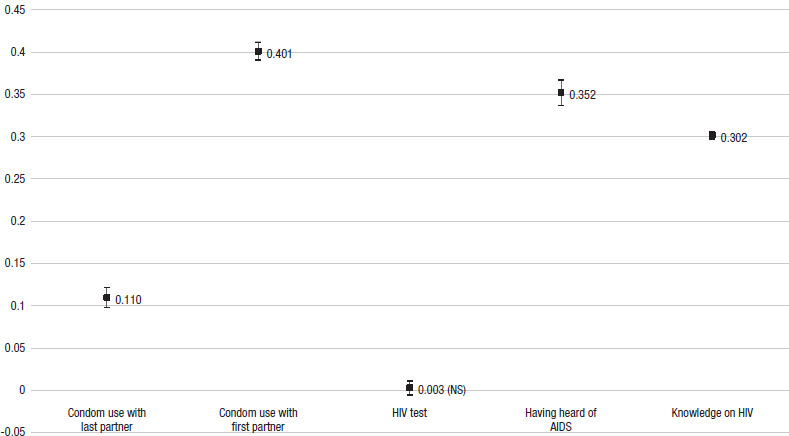
Concentration index as a measure of health inequalities for HIV/AIDS analyzed indicators in LAC using per-capita GDP-PPP

***Source:*** Authors based on UNICEF Multiple Indicator Cluster Surveys (MICS) & Demographic and Health Surveys Program (DHS) surveys, as well as World Bank per-capita gross domestic product adjusted by purchase power parity (pc-GDP-PPP)

In figure 1, we present the inter-country CI for analyzed indicators (with their confidence intervals); as reported, while there is no difference in the probability of testing related to the per-capita GDP-PPP, knowledge of AIDS and HIV was higher among countries with higher per-capita GDP-PPP, as well as condom use, both with the last and first sexual intercourse (condom use with first sexual partner was not included in the intra-country analysis as only 14 of the 20 country surveys included this indicator).

## DISCUSSION

In a region known by its social inequality, adolescents and young females in Latin America and the Caribbean face an heterogenous risk for HIV that is related to their household socioeconomic conditions and their country economic development. In this analysis that includes data from 20 countries comprising 57 % of total population of females 15 to 24 years in the region, we reported intra- and inter-country inequalities on indicators that reflect risk for HIV; young women age 15 to 24 living in countries with higher GDP-pc have greater knowledge about HIV and higher probability of using condoms in first and last sexual intercourse. In addition, analyzed countries with higher socioeconomic status (measured using a wealth index) young women reported greater HIV knowledge and higher probability of using condoms.

Health inequalities in the region have been already documented for different health outcomes, the role of social determinants of health in perpetuating and even widening gaps between those that are better off and those living in poor households are well known (30). Even in countries with lower social inequality – such as Cuba or Uruguay – there is evidence of inequalities in the analyzed indicators.

Moreover, it is important to highlight that even countries with better performance on the selected indicators are far from optimal levels. The best performance for condom use in the more recent sexual intercourse is 57.3 %, so in the best situation 4 out 10 females reported not using protection to avoid STI.

While the reported results identify a relevant gap on the analyzed indicators, further research is needed to better understand the process that have produced those differences. The identified inequalities are the result of social determinants of health that could materialize in different ways.

While the HIV prevalence in the region, compared to other regions, is in general low the socio-economic circumstances where young women live greatly affect their access to HIV prevention interventions, highlighting the need to address social inequalities that contribute to the persistence of new HIV infections in the region (31).

The conjunction of gender, age and poverty is reflected in inequalities in knowledge – relevant for prevention – and in access to prevention interventions. In this analysis, correct knowledge of HIV transmission were higher among individuals in households from higher socioeconomic status. Adolescent girls and young women from poor household have imperfect or incomplete information and less likely to have the means to protect themselves.

This is reflected in reported condom use in the last sexual intercourse. The distribution is pro-rich for most countries in the region. Use of condom in the last sexual intercourse reflects access as well as the ability to negotiate and willingness to use it.

These results are similar to estimations from South Africa, where knowledge and condom use were also pro-rich distributed, while for LAC the CI present higher values (32, 33).

A previous analysis from Mexico has also reported a higher condom use among adolescents from affluent households, which is consistent with the results reported here (15).

To our knowledge, this is the first regional analysis on HIV related inequalities affecting adolescents and youth women. There are limitations to this analysis. We used data from two set of surveys that are similar but no identical in terms of questionnaires and procedures. These surveys are only available for 20 out of 36 countries in the region. However, both sets are household probabilistic surveys, and we use comparable questions that produce the same set of indicators. Not all the countries in the region are included in the analysis as not all countries participate in either MICS or DHS, but countries included comprise 57 % on the population of interest. Data were not available for Brazil, the largest country in the region. It is possible that the distribution of the analyzed variables is different for other countries not included here. For the inter-country analysis, we included surveys within a 10 year window, and the findings could reflect changes over time and no actual differences between countries. By using age-standardized indicators we minimized the bias due to changes in age distribution across countries. As the surveys used for the analysis are multi-purpose surveys, we were not able to estimate all desirable indicators in terms of measuring inequalities on HIV indicators, so our analysis is limited to available indicators.

The limited number of indicators used highlights the scarcity of HIV related data available on adolescents and young women in the region. There is a need to improve the generation of evidence and data available for this population; moreover, it is equally important to generate evidence on adolescents and young people from key population (34).

### Conclusions

We report inequalities in variables that are relevant to the goal of achieving zero HIV transmission. To accomplish this goal, it is not sufficient to implement effective interventions to increase knowledge and improve behaviors. Such interventions need to target economically vulnerable populations in order to close the existing inequity gaps.

Acknowledging the multi-factor process behind limited condom use, increasing access to condoms by freely distributing them at schools and other public venues should be implemented together with comprehensive sexuality education to strengthen decision-power abilities, and economic opportunities for young females that could change their life perspectives. Access to secondary education is the most cost-effective approach to promote adolescent health and the use of conditional cash-transfer programs has proved successful in maintaining girls in schools and reduce the risk of HIV transmission (35). Access to comprehensive sexuality education may increase HIV knowledge among those in the socioeconomic vulnerable groups and could be useful to increase HIV testing overall but in particular among those at higher risk.

Economically disadvantage adolescents and young women in LAC face an increased risk for HIV, as they are less aware of HIV and how it is transmitted and less likely to use a condom with their sexual partners. There is an urgent need to review the HIV and STI prevention interventions for young women and girls to ensure they are sensitive to their socioeconomic context.

## Availability of data and materials.

All data used for this analysis are available at the DHS and MICS websites with registration, as well as at the World Bank indicators web site. Pooled data for this analysis as well as the Stata code are available at https://figshare.com/articles/dataset/Data_for_Socioeconomic_inequalities_HIV_Adolescents_LAC/12251981

## Disclaimer.

Authors hold sole responsibility for the views expressed in the manuscript, which may not necessarily reflect the opinion or policy of the *RPSP/PAJPH* and/or PAHO.
